# Factors and outcomes associated with the induction of labor in referral hospitals of Amhara regional state, Ethiopia: a multicenter study

**DOI:** 10.1186/s12884-021-03709-5

**Published:** 2021-03-20

**Authors:** Tibeb Zena Debele, Endeshaw Admassu Cherkos, Marta Berta Badi, Kiber Temesgen Anteneh, Fitsum Wolde Demssie, Abdella Amano Abdo, Muhabaw Shumye Mihret

**Affiliations:** 1 Department of Clinical Midwifery, School of Midwifery, College of Medicine and Health Sciences, University of Gondar, PO. Box 196, Gondar, Ethiopia; 2 Department of Women’s and Family Health, School of Midwifery, College of Medicine and Health Sciences, University of Gondar, Gondar, Ethiopia; 3 Department of Midwifery, Arbaminch College of Medicine and Health Science, Arbaminch University, Arbaminch, Ethiopia; 4 Department of Epidemiology and Biostatistics, College of Medicine and Health Sciences, Hawassa University, Hawassa, Ethiopia

**Keywords:** Amhara regional state, Failed induction, Induction of labor, Referral hospitals

## Abstract

**Background:**

Induction of labor is an artificial initiation of uterine contractions after fetal viability with the aim of vaginal delivery prior to the onset of spontaneous labor. Prevalence of induction of labor is increasing worldwide with subsequent increase in failure rate. However, there is limited evidence on labor induction in Ethiopia. Therefore, this study was aimed at assessing the prevalence and associated factors of failed induction of labor among women undergoing induction of labor at referral hospitals of Amhara national regional state, Ethiopia, 2016.

**Method:**

A multicenter cross-sectional study was conducted at referral hospitals found in Amhara national regional state from February 01 to September 30, 2016. Multistage sampling technique was employed to select a total of 484 women who underwent labor induction. Pre-tested structured questionnaires and checklists were used to collect the data. Data were entered into EPI info version 7 and analyzed using SPSS version 20 software. Stepwise Binary Logistic regression model was fitted to identify factors associated with failed induction of labor. The level of significance was determined based on the adjusted odds ratio with 95% confidence interval at the *p*-value of ≤0.05.

**Result:**

The prevalence of failed induction of labor among women undergoing induction of labor was 31.4% (95% CI: 27.0, 36.0). Failed induction of labor was independently predicted by a Bishop score of ≤5 (AOR = 2.1; 95% CI: 1.3, 3.6), prolonged latent first stage of labor (AOR = 2.0; 95% CI: 1.2, 3.5), induction with oxytocin alone (AOR = 4.2; 95% CI: 2.2, 8.1), nulliparity (ARO = 1.9; 95% CI: 1.2, 2.9), post term pregnancy (AOR = 4.1; 95% CI: 1.8, 9.3) and hypertensive disorder of pregnancy (AOR = 2.4; 95% CI: 1.5, 5.1).

**Conclusion:**

Failed induction of labor was high in the study area compared to the reports of previous studies done in Ethiopia. The majority of the determinants of failed induction of labor were connected with unjustifiable and inconsistent indication of induction of labor. Thus, preparing standardized practical guidelines and preventing unjustifiable case selection may help reduce the current high failure rates.

**Supplementary Information:**

The online version contains supplementary material available at 10.1186/s12884-021-03709-5.

## Background

Maternal and child health issues are among the top public health concerns globally [1]. The Ethiopian government is working towards improving maternal and neonatal health and establishing measures to ensure that no mother dies in the process of giving birth [2]. In this regard, effective obstetric management is crucial, and obstetric management efforts need to be rigorous so as to overcome the incidence of poor outcomes. Therefore, inducing labor and improving the outcomes of labor in those women for whom spontaneous labor is not eminent is an integral part of obstetric management efforts [3].

Induction of labor (IOL) is the artificial initiation of uterine contractions after the period of viability with the intention of accomplishing vaginal delivery prior to onset of spontaneous labor [4]. The World Health Organization (WHO) recommends that IOL needs to be indicated primarily for the improvement of quality of care and outcomes for pregnant women undergoing the procedure in under-resourced settings [5]. Globally, IOL brings about increasing incidence of failed IOL. Of course, the definition of failed IOL is inconsistent across the literature and so far no consensus has been reached about its definition [3, 4]. A variety of end points such as mode of delivery, vaginal delivery within a certain time interval or achievement of active phase of labor have been suggested. Therefore, comparison becomes difficult due to the existing heterogeneity within the literature [6]. In general, a definition of failed IOL that coincides with mode of delivery seems the best option since it evaluates the accomplishment of the purpose of induction [7].

Induction of labor (IOL) is not without risks and it is physically uncomfortable for many women [5]. Although a 2018 New England Journal of Medicine article showed that elective induction at 39 weeks reduces cesarean sections (C/S), in general terms, the risk of C/S is doubled with IOL compared to spontaneous onset of labor [8, 9]. The rising number of medical and obstetrical indications of IOL as well as marginally indicated and elective inductions contribute to the increased rate of failed IOL [10]. The increased risk of C/S following IOL has been associated with higher rates of fetal and maternal morbidity [11, 12]. Failure of IOL is a clinical and public health concern as it results in increased C/S rate which is a classic example of the mismatch between evidence and practice in obstetrics. Poor IOL outcomes are rising making it a priority area in maternal health [4, 13].

Conducting empirical studies on the determinants of failed IOL would help improve the success rate of IOL. Existing evidence points out that the failure rate of IOL is increasing worldwide. However, evidence regarding prevalence and associated factors of failed IOL in Ethiopia is scarce. To the best of the investigators’ knowledge, there was no prior investigation study performed on IOL in the study settings.

Therefore, this study assessed the prevalence of failed IOL and associated factors among women who underwent labor induction at referral hospitals found in Amhara national regional state, Ethiopia. The information generated from the current study may help clinicians to select appropriate patients and method of induction in order to achieve successful outcomes for the mother and her newborn.

## Methods

### Study design, period and settings

A multicenter cross-sectional study was conducted at referral hospitals of Amhara national regional state from February 01 to September 30, 2016. Amhara national regional state is serviced by 19 hospitals, 220 health centers, and 2941 health posts. In this region, there are Five referral hospitals namely Gondar University Comprehensive Specialized Hospital (GUCSH) Felege Hiwot referral hospital (FHRH), Dessie referral hospital (DRH), Debremarkos referral hospital (DMRH), and Debre Birhan referral hospital (DBRH). Three out of five (60%) of the referral hospitals (i.e., GUCSH, DMRH and DBRH) were selected randomly. Each referral hospital is assumed to offer services for 5 million people, has 100–200 beds, 2000–3000 deliveries per year, and 5–8 deliveries per day [14]. Regarding the composition of the team of health care professionals, the maternity wards of each hospital have BSc and MSc educated midwives as well as intern, resident, and specialist doctors in Obstetrics and Gynecology. Moreover, there is an intra- and inter- profession consultancy system. In addition, there is a registry of obstetrics where all gynecologic, obstetric and neonatal related data are registered daily.

### Source and study population

The source population for this study was pregnant women who underwent IOL at referral hospitals in Amhara national regional state. Therefore, we included women who underwent IOL at the selected referral hospitals of Amhara national regional state during the data collection period. We excluded mothers who had neither a reliable due date nor any ultrasound measurement records. In addition, women who had serial IOL (i.e., a second or third attempt at IOL after the first full phases of IOL were completed with no initiation of labor) were excluded.

### Sample size determination and sampling technique

A single population proportion formula was employed to calculate the sample size (N) by considering the following statistical assumptions: magnitude of failed induction - 17.3% (i.e., *p* = 0.173) taken from a previous study done at Hawassa referral hospital [15]; level of significance - 5% (α = 0.05), Z α/2–1.96; margin of error - 5% (d = 0.05); design effect - 2, and non - response rate − 10%.

Accordingly, N= $$ \frac{{\left({\mathrm{z}}_{\frac{\upalpha}{2}}\right)}^2\mathrm{p}\left(1-\mathrm{p}\right)}{{\mathrm{d}}^2}=1.962\ast 0.173\left(1-0.173\right)/0.052=3.8416\ast /0.0025=220 $$.

Thus, after multiplying by a design effect of 2 and adding 10% non response rate, the final sample size obtained was 484.

We employed a two-stage sampling technique. First, we selected three hospitals out of the five referral hospitals randomly. Then, we utilized a systematic random sampling technique to select the study participants at the selected hospitals. The total sample size was proportionally allocated for the three selected referral hospitals (i.e., GUCSH, DMRH and DBRH) depending on their load of induction cases. The selection interval (i.e., k-interval) was yielded by dividing the total number of induction cases in the three hospitals on a yearly basis (i.e., 727) by the calculated sample size of the study (i.e., 484). Hence, k - interval = 727/484 = 1.5 ≈ 2. The first participant was selected randomly from the first two induction cases and then every second case was selected. If an individual did not satisfy the inclusion criteria, the next participant was selected.

### Variables of the study

This study poses one outcome variable and a large number of explanatory variables. Specifically, a failed IOL is the outcome variable, whereas socio-demographic variables (age, ethnic group, marital status, educational level, residence, religion, mother’s occupation); obstetric variables (bishop score, gestational age, number of parity, poor obstetric history, birth weight, duration of latent stage of labor); indications (PROM, fetal death, Hypertensive Disorder of Pregnancy (HDP), post term (≥42 completed weeks), growth restriction, medical condition), and induction (type, dose) are the independent variables of this study. In addition, we recorded other variables in the descriptive section. These variables included mode of delivery such as non-assisted vaginal delivery, instrumental vaginal delivery (i.e., vacuum and /or forceps delivery) and C/S, and indications for C/S like non-reassuring fetal heart rate (i.e., fetal heart beat out of the range of 120–160 beats per minute) and failure to achieve first stage of active labor.

### Operational definitions

**Failed induction of labor**: women who had induction of labor and delivered the fetus through C/S [7].

**Poor obstetrics history**: if a woman had experienced any of the following events on two or more occasions in the past: consecutive spontaneous abortions, early neonatal deaths, stillbirths, intrauterine fetal deaths, intrauterine growth retardation, congenital anomalies in the fetus [16].

**Bishop score**- a score ≤ 5 represents an unfavorable cervix, whereas a score > 5 indicates a ripe cervix [17].

**Prolonged Latent First Stage of Labor (PLFSL)**: latent first stage of labor lasting more than 18 h [18].

**Instrumental delivery**: we used the term instrumental delivery to express either vacuum or forceps delivery.

**Hypertension:** maternal hypertension was considered when any record of maternal blood pressure of ≥140/90 mmHg was obtained during pregnancy and child birth [19].

**Fetal distress**: Normal fetal heart rate pattern was defined as a baseline heart rate of 110–160 beat per minute with a variability of 5–25 beats per minute and no repetitive decelerations. All findings which deviated from this normal fetal heart rate pattern definition were considered as fetal distress [20].

**Post term pregnancy:** pregnancy lasting ≥42 completed weeks (i.e., 42 0/7 weeks of gestation and beyond [21].

### Data collection tools and procedures

Data were collected by face-to-face interview using a structured, pre-tested questionnaire (Additional file 1) to assess the socio-demographic characteristics of the mothers and a checklist was used to collect the data regarding the induction process. In other words, we utilized two data collection tools – the questionnaire and the checklist. The questionnaire was provided in local language (Amharic) through interviewer administered approach and applied on the study participants (mothers), whereas, the checklist was provided in English and was filled by the data collectors through chart (document) review. The tools used in our study did not require a license to administer. The questionnaire was prepared in English and translated into the local (Amharic) language and back to English. Three diploma midwives were used to collect the data. A BSc midwife from each of the three hospitals was assigned to supervise the data collection process.

### Data quality control

The quality of data was assured by proper design and pre-testing of the questionnaires in Felegehiwot hospital on 48 mothers. Training was given for data collectors and supervisors before the actual data collection. After interviewing the study participants (i.e., women who had induction of labor), charts and registration books were revised to improve the quality of the data. Every day after data collection, the questionnaires were reviewed and checked for completeness and consistency by the supervisors and the principal investigator and necessary feedback was offered to data collectors the next morning. Epi info version 7 was used to minimize data entry error.

### Data processing and analysis

The completed questionnaires were checked, coded and entered into Epi Info version 7 and exported to SPSS version 20 software. Descriptive statistics like percentage, mean and standard deviation (SD) were used for the presentation of demographic data and prevalence of failed IOL. Tables were employed for data presentation.

Binary logistic regression model was used to identify factors associated with failed IOL. Variables with *p*-value less than or equal to 0.20 were fitted into multivariable logistic regression models to control for the possible effect of confounders and finally the variables which had independent association with failed induction were identified on the basis of AOR with 95% CI at *p*-value of ≤0.05.

## Results

### Socio-demographic characteristics

During the study period, 969 mothers underwent IOL, of which 484 were randomly sampled for inclusion as described in the methods. The mean age of respondents was 26.9 years with standard deviation (SD) of 4.7, and 405 (83.7%) of the mothers were below 30 years. More than two-thirds of the study participants were urban residents **(**Table 1**).**

**Table 1:** Socio demographic characteristics of mother who undergo induction of labor in Amhara regional state referral hospitals, Northwest Ethiopia, 2016 (*N* = 484)

Variables	Number	Percent
Age of the mothers at interview (in year) (mean, SD,26.89 ± 4.68)		
18–30	405	83.7
> 30	79	16.3
Marital status of the mothers		
Single	7	1.4
Married	476	98.4
Divorced	1	0.2
Religion of the mothers		
Orthodox	358	74
Muslim	121	25
Protestant	5	1
Ethnicity of the mothers		
Amhara	437	90.3
Tigre	15	3.1
Oromo	32	6.6
Educational status of the mother		
Unable to read and write	125	25.8
Able to read and write	28	5.8
Primary educations	120	24.8
Secondary education and above	211	43.6
Occupational status of the mothers		
House wife	198	40.9
Governmental employee	93	19.2
Farmer	65	13.4
Merchant	54	11.2
Self-employee	42	8.7
Others	32	6.6
Residence		
Urban	334	69
Rural	150	31

### Obstetrics characteristics of respondents

Two hundred and forty-eight (51.2%) of the respondents were multiparous and about 340 (70.2%) of the IOL procedures were undertaken at the GA of 37–41 completed weeks. More than half (54.3%) of the participants had a pre-induction bishop score of > 5 and one - tenth (10.8%) of the respondents had a poor obstetric history. Three hundred and sixty-seven (75.8%) of laboring mothers spent less than 18 h in the latent first stage of labor **(**Table 2**).**

**Table 2:** obstetric characteristics of mothers who undergo induction of labor in Amhara regional state referral hospital, North West Ethiopia,2016(*N* = 484)

Variables	Number	Percent
Parity		
Nulliparous	236	48.8
Multiparous	248	51.2
Gestational age		
28–36	61	12.6
37–41	340	70.2
≥ 42	83	17.2
Pre-induction bishop score		
> 5	263	54.3
≤ 5	221	45.7
Duration of latent first stage of labor		
> 18 h	117	24.2
≤ 18 h	367	75.8
Poor obstetric history		
Yes	96	19.8
No	388	80.2

### Outcome, method and indication for induction

The proportion of mothers experiencing failed IOL was found to be 31.4% (95% CI: 27, 36). Regarding the routes of delivery, about 212 (43.8%), 120 (24.8%) and 152 (31.4%) of the labor induced mothers underwent non-assisted vaginal delivery, instrumental vaginal delivery and C/S respectively.

About 58.6, 29.1 and 12.3% of the induction procedures were achieved using oxytocin alone, prostaglandins alone and combinations of different methods of induction respectively.

Non-reassuring fetal heart rate (57.2%), failure to achieve active first stage of labor (36%) and other factors (7%) accounted for C/S after an attempt of IOL.

The leading indication of IOL was HDP (35.5%) followed by PROM (34.5%), post term (16.3%) and others (13.6%).

### Factors associated with failed induction of labor

Both bivariate and multivariable logistic regression analyses have been done. According to the result of multivariable logistic regression analysis, IOL with oxytocin alone (AOR = 4.2;95% CI: 2.2,8.1), nulliparity (AOR = 1.9; 95% CI:1.2,2.9), PLFSL (AOR = 2.0; 95% CI:1.5,3.5), Bishop score of ≤5 (AOR = 2.1; 95% CI: 1.3,3.6), post term pregnancy (AOR = 4.1; 95% CI: 1.8,9.3) and HDP (AOR = 2.4; 95% CI: 1.5,5.1) are significantly associated with failed IOL (Table 3).

**Table 3:** Bivariate and multivariable logistic regression analysis of factors associated with failed induction of labor among mothers who had induction of labor in Amhara regional state referral hospitals, North West Ethiopia, 2016(*N* = 484)

Variables	Failed IOL		COR(95%CI)	AOR(95%CI)
	YES	NO		
**Methods of induction**				
Oxytocin	101	183	1.3 (0.8,2.0)	**4.2 (2.2,8.1)**
Either combination	10	49	0.5 (0.2,1.0)	0.4 (0.2,0.9)
prostaglandins	42	99	1.00	1.00
**Parity**				
Nulliparous	92	144	2.0 (1.4,2.9)	1.9 (1.2,2.9) **
Multiparous	61	187	1	
**Gestational age**				
≤ 36	9	52	1.00	*
37–41	112	228	2.8 (1.4,6.0)	
≥ 42	32	51	3.6 (1.6,8.4)	
**Indication**				
Post term	30	49	2.8 (1.3,6.0)	4.1 (1.8, 9.3) **
PROM	44	123	1.6 (0.8,3.3)	0.9 (0.4, 2.0)
Hypertensive disorder	67	105	2.9 (1.4,5.8)	**2.4 (1.5, 5.1)**
Other	12	54	1.00	
**Age**				
≤ 30	139	266	1.00	*
> 30	14	65	0.4 (0.2,0.8)	
**Duration of induction**				
> 18 h	44	73	1.4 (0.9,2.2)	**2.0 (1.5,3.5)**
≤ 18 h	109	258	1.00	
Previous obstetric complications				
Yes	131	257	0.6 (0.4,1.0)	
No	22	74	1.00	*
**Bishop score**				
≤ 5	78	143	1.4 (0.9,2.0)	**2.1 (1.3,3.6)**
> 5	75	188	1.00	
**Birth weight**				
< 4000 g	12	30	1.00	*
≥ 4000 g	141	301	0.9 (0.4,1.7)	

* Not significant in back ward stepwise logistic regression

**P –value ≤0.001

others-IUFD, DM, IUGR, postdate, oligohydramnios

## Discussion

Labor induction is one of the fastest growing medical procedures. Studies done in America show a nationwide twofold increase in the rates of IOL between the late eighties and early nineties [22]. Clinicians are concerned over the inconsistent indications for and increased rates of induction. Moreover, there was dearth of evidence regarding the magnitude and predictors of failed IOL in Ethiopia and in the study settings particularly. Thus, this study was conducted to provide valuable inputs on IOL so that stakeholders can use the information to improve pregnancy outcomes following IOL, and to inspire further research in this field. The study exhibited that failed IOL was high and can be predicted by an unfavorable pre induction Bishop Score, PLFSL, induction with oxytocin alone, nulliparity, post term pregnancy and HDP. This study shows that the prevalence of failed IOL in the study settings was 31.4%, which is in line with a WHO study report in eight Latin American countries − 30% [23] and a result which was reported at Kathmandu Medical College - 34.6% [24]. However, the magnitude of failed IOL found in this study was higher than the study done at Jimma University - 21% [25] and Aga Khan Hospital - 18.1% [26]. This difference might be due to the variances in the selection criteria in which the previous studies defined failed induction only if mothers failed to achieve active first stage of labor after 6 to 8 h. In this study, any labor that led to C/S after initiation of labor induction was considered a failed induction regardless of the time. Another factor that could lead to higher rates is that our study included all gestational ages while the previous studies included only term and post term pregnancies. Thus, the higher success rate of IOL in the previous studies could be explained by maturity of cervix as the cervix ripens when the pregnancy approaches term. The current study showed that the odds of failed IOL were 2.5 times higher in mothers with a Bishop score of ≤5 compared with the reference group. This study is in line with studies done in different geographical areas [6, 27, 28]. The statistically significant association of failed IOL and unfavorable Bishop Score is plausible as an unfavorable Bishop score is a sign of non-effaced cervix that may result in failure to achieve first stage of labor. However, the study done at Meir Hospital in Israel showed that there was no association between success of induction and pre-induction Bishop score ≤ 5 [29]. This difference could be due to the fact that the study participants were only multiparous women whose cervices efface easily but, in our study, both nulliparous and multiparous women were included.

In our study, women with PLFSL were 2.3 times more likely to experience failed IOL as compared to those women with shorter duration. This finding is also supported by previous studies done in different settings [26, 30]. This might be due to the fact that prolonged duration of labor can increase the risk of adverse outcomes such as meconium-stained amniotic fluid which in turn can bring about delivery through C/S.

The result of the final model analysis in this study also shows that mothers who had IOL with oxytocin alone were more likely to face failed IOL. This is in line with a study done in California [31]. This could be due to the fact that oxytocin is mainly responsible for uterine contraction and providing this uterotonic agent at unfavorable cervical status might lead to failure to achieve active first stage of labor. In this regard, failure in achieve active first stage of labor is one of the common indications of C/S as it is observed even in our study that about more than one-third (36%) of the C/S procedures were done because of this indication. On the other hand, a study conducted in Iran showed there was no difference in rate of failed IOL when using oxytocin [32]. This difference might be due to the difference in selection criteria of study participants which included mothers who had gestational age of ≥37 weeks who probably have a favorable Bishop. In addition, in our hospitals, there is relatively shorter duration of oxytocin administration (6–8 h) compared with Bahonar Hospital, where it is a minimum of 17 h. It is evident from literature that a longer duration of oxytocin administration has a good success rate.

Maternal parity was also another important determining factor for failed IOL. Nulliparous mothers were 1.9 times more likely to have a failed IOL. This result is supported by different literature [33, 34]. This is consistent with the theory that the primiparous cervix is immature, and an immature cervix requires more time to stimulate through induction. On the contrary, a study done in California [35] showed that there was decreased odds of failed IOL among nuliparas.

The likelihood of failed IOL was1.8 times higher among mothers who had induction of labor for the indication of HDP. This is in line with a study done at the University of Washington [36]. This might be due to the reality that HDP predisposes fetal compromise as a result of utero -placental insufficiency. In addition, it may necessitate preterm labor induction before maturation of the fetus which may easily lead to a non-reassuring fetal heart beat pattern when uterotonic agents are administered.

The odds of failed IOL were 4.1 times higher among mothers who had IOL for the indication of post term pregnancy. This is in line with the study done at Hawassa, Ethiopia [15]. The association can be explained through placental calcification at post term pregnancy. The placenta can become calcified and less functional as the pregnancy approaches post term. As a result, the flow of oxygen and other nutrients to the fetus is compromised which leads to less ability to cope with uterotonic agents. On the contrary, a study done in Copenhagen showed that there was no difference in failed induction rate when comparing IOL in a specific gestational week from week 39 with a later labor. This difference could be due to the fact that we used maternal last normal menstrual periods as well as ultrasound estimations at every gestational age. This may lead to an inaccurate estimation, as it is known that first trimester ultrasounds are most accurate in determining GA. In addition, our study excluded women who had C/S for complications of pregnancy and not as a consequence of induction which may significantly affect the result.

### Limitations

Factors such as clinician practice during IOL were not included.GA was determined by LNMP and ultrasound measures done at any time which might show slight inaccuracy compared with the result of early ultrasound measurement. Another important issue that readers need to consider is that the high prevalence of failed IOL in our study could be attributed to C/S procedures indicated for non-reassuring fetal heartbeat rather than failed labor as the study lacked a control group.

## Conclusion

The prevalence of failed IOL among mothers undergoing labor induction was relatively high in the study settings compared with previous studies in the country. Failed IOL was independently predicted by prolonged latent first stage of labor, unfavorable Bishop score, post term delivery, nulliparity, HDP, and induction of labor with oxytocin alone. The majority of the determinants of failed induction of labor were connected with unjustifiable and inconsistent indication of induction of labor. Therefore, the authors would like to convey the following recommendations: an evidence-based clinical guideline to enhance successful IOL outcome needs to be developed to standardize the induction protocol in all hospitals; appropriate case selection should be employed when starting the induction process and borderline indications should be wait until spontaneous labor has started; patients with poor Bishop score need to be given an option of an elective C/S after the initial process of cervical ripening; and further randomized Clinical Trial (RCT) studies need to be conducted on labor induction so as to generate further evidence for improving pregnancy outcomes.

## Supplementary Information

**Additional file 1.**

## Data Availability

The datasets used and/or analysed during the current study available from the corresponding author on reasonable request.

## Abbreviations

C/S: Caesarean section
DMRH: Debremarkos referral hospital
DM: Diabetes miletus
FRH: Felegehiwot referral hospital
GA: Gestational age
GUCSH: Gondar university comprehensive specialized hospital
HDP: Hypertensive disorders of pregnancy
IOL: Induction of labor
IUFD: Intra uterine fetal death
IUGR: Intra uterine growth restriction
LNMP: Last normal menstrual period
PLFSL: Prolonged latent first stage of labor
SPSS: Statistical package for social sciences
WHO: World health Organization
