# Supplementary material for: Factors and outcomes associated with the induction of labor in referral hospitals of Amhara regional state, Ethiopia: a multicenter study
Source: BMC Pregnancy Childbirth. 2021 Mar 20;21:225. doi: 10.1186/s12884-021-03709-5 (PMC8095340; doi:10.1186/s12884-021-03709-5)
Supplement: Supplementary file 1 — Additional file 1. [file 12884_2021_3709_MOESM1_ESM.docx]

Annex 1: English Version Questionnaire

Questionnaire is prepared to assess factors and outcomes associated with the induction of labour in referral hospitals of Amhara regional state, Ethiopia.

Hello! My name is -----------------------------------I am one of the members of the research team. The purpose of this questionnaire is to gather information on factors associated with failed induction of labor.

I have identified you as a study participant hoping that you would be willing to help me by providing information. I have several questions which I would like to ask you, if you have the time and are willing. All information you provide will be kept confidential. I will not include any identifiers, such as your name or exact address. Only honest answers would contribute to improvement of health planning. Your role in the success of the research is important and I appreciate your contribution to the research. Would this be okay with you?

I understood about the advantage of the research and the roles I will have in the research. I have agreed to participate in the research.

A. Yes

B. No

If respondent agrees to be interviewed, remind them to put the signature:

Signature of the respondents______________________________

- Starting time___________
- End time_________

001. Questionnaire Code ________

Date of data collection----------------------------------------

Name of data collector--------------------------------------- signature--------------------

Name of supervisor------------------------------------------- signature----------

Instruction: for each of the following questions please circles the number of alternative/s that fit for your response or fill the blank space.

Part I: Demographic and baseline health information

| No | | Questions | Answers | Skip to Qn No--- |
| --- | --- | --- | --- | --- |
| 101 | | How old are you? | _________years |  |
| 102 | | Marital status | 1. Married 2. Divorced 3. Widowed 4. Single 5. Separated 6. Others specify________ |  |
| 103 | | Religion | 1. Orthodox 2. Muslim 3. Protestant 4. Other, specify________ |  |
| 104 | | Ethnicity | 1. Amhara 2. Oromo 3. Tigre 4. Other, specify_______ |  |
| 105 | What is your educational level? | | 1. Unable to read & write 2. Can read and write 3. Primary education (1-8) 4. Secondary education & above |  |
| 106 | What is your occupation? | | 1. Housewife 2. Merchant 3. Farming 4. Government employed 5. Daily laborer 6. Other specify________ |  |
| 107 | Residence | | 1. Urban 2. Rural |  |

Part II: Data on Induction of labor

| Sr. No | Questions | Answers | Skip to qn. No. |
| --- | --- | --- | --- |
| 201 | Time of inductionstarted |  |  |
| 202 | Indication(s) for Induction, tick where applicable(more than one answer is possible) | 1.Post term  2.PROM  3.Hypertensive disorders  4.Diabetes  5.IUGR  6.Others specify____ |  |
| 203 | Method of induction (indicate)(more than one answer is possible) | 1.Amniotomy  2.Intravenous pitocin infusion  3.Prostaglandins  4.Balloon catheter |  |
| 204 | If misoprostol only, route of  administration | 1.Vaginal  2.Oral  3.Sublingual |  |
| 205 | misoprostol dose | 1.25  2.50ug  3.100ug  4.150ug  5.200ug  6.>200ug |  |
| 206 | oxytocine dose |  |  |
| 207 | Length latent first stage of labor | _________hours |  |

Part III: obstetric factor

| Sr. No | Questions | Answers | Skip to Qn No. |
| --- | --- | --- | --- |
| 301 | Parity |  |  |
| 302 | Previous obstetric complications | 1.yes  2.no |  |
| 303 | If yes specify……. |  |  |
| 304 | Fetal birth weight in KG |  |  |
| 305 | Gestation age in weeks (indicate) |  |  |
| 306 | Bishop’s score in umber | ­­­­­______________number |  |
| 307 | Reasons for caesarian section | 1. Fetal distress  2.Failure to achieve active1st stage of labor  3.others______________ |  |

THANKS A LOT!!!
